# Immunoscore^®^ as a predictor of response to chemotherapy in stage II and stage III colon cancer

**DOI:** 10.1186/2051-1426-3-S2-P89

**Published:** 2015-11-04

**Authors:** Bernhard Mlecnik, Anne Berger, Franck Pages, Jerome Galon

**Affiliations:** 1 INSERM, Paris, France; 2 APH-HP, Paris, France

## Background

No biomarker has been validated so far to predict the need for adjuvant chemotherapy in Stage II colon cancer (CC), and current guidelines, e.g. NCCN, only rely on the identification of high-risk features such as MSI, pathological tumor characteristics or expected chemotherapy efficacy/safety ratio in each individual patient. In contrast, adjuvant chemotherapy is recommended for all Stage III colon cancer (CC). The Immunoscore^®^ (IS^®^), based on CD3- and CD8-positive cell quantification using a digital pathology automated analysis, has been shown to be a strong prognostic indicator in CC, surpassing the TNM classification [1]. We explore here the potential of the Immunoscore^®^ to predict the need for 5-FU adjuvant chemotherapy in Stage II and in Stage III CC.

## Methods

The IS^®^ was calculated as previously described on a retrospective cohort of 456 colorectal cancer patients from Hôpital Européen Georges Pompidou, who underwent primary tumor resection between 1990 and 2003. For the analysis of chemotherapy benefit on disease-free survival, patients were grouped in 3 subcohorts based on their IS^®^: IS^®^ 0-1 (worst prognosis), IS® 2-3 (intermediate prognosis) and IS^®^ 4 (best prognosis).

## Results

Stage II and Stage III cases represent resp. 327 (71.7%) and 129 (28.3%) of the cohort. 5-FU adjuvant chemotherapy was administered to 13.8% and 60.5% of Stage II and Stage III CC resp. IS^®^ 0-1, IS^®^ 2-3 and IS^®^ 4 represented 11.6%, 35.2% and 53.2% in the Stage II population, and 18.6%, 36.4% and 45% of patients in the Stage III population. Disease-free survival of IS^®^ 2-3 patients was improved by administration of 5-FU chemotherapy (Stage III: HR=2.69 (1.02-7.11) pV = 0.038, and Stage II: HR=1.47 (0.35-6.18) pV not reaching significance). IS^®^ 4 patients had the best and IS® 0-1 patients the worst outcome in both Stage II and Stage III populations. There was no statistically significant benefit of chemotherapy on disease-free survival for IS^®^ 4 or IS^®^ 0-1 patients in both Stage II and Stage III populations.

## Conclusion

IS^®^, which has been developed to be an easy-to-use routine practice biomarker, may represent a valid biomarker to precisely identify stage II and stage III CC patients most likely to benefit from 5-FU chemotherapy. These results, based on a single center retrospective cohort, warrant further validation on a larger, clinical trial, cohort.

